# The probability of genetic parallelism and convergence in natural populations

**DOI:** 10.1098/rspb.2012.2146

**Published:** 2012-10-17

**Authors:** Gina L. Conte, Matthew E. Arnegard, Catherine L. Peichel, Dolph Schluter

**Affiliations:** 1 Biodiversity Research Centre and Zoology Department, University of British Columbia, Vancouver, British Columbia, CanadaV6T 1Z4; 2 Division of Human Biology, Fred Hutchinson Cancer Research Center, Seattle, WA 98109, USA

**Keywords:** parallel evolution, convergent evolution, genetic parallelism, genetic convergence, genetic biases and constraints, genetics of adaptation

## Abstract

Genomic and genetic methods allow investigation of how frequently the same genes are used by different populations during adaptive evolution, yielding insights into the predictability of evolution at the genetic level. We estimated the probability of gene reuse in parallel and convergent phenotypic evolution in nature using data from published studies. The estimates are surprisingly high, with mean probabilities of 0.32 for genetic mapping studies and 0.55 for candidate gene studies. The probability declines with increasing age of the common ancestor of compared taxa, from about 0.8 for young nodes to 0.1–0.4 for the oldest nodes in our study. Probability of gene reuse is higher when populations begin from the same ancestor (genetic parallelism) than when they begin from divergent ancestors (genetic convergence). Our estimates are broadly consistent with genomic estimates of gene reuse during repeated adaptation to similar environments, but most genomic studies lack data on phenotypic traits affected. Frequent reuse of the same genes during repeated phenotypic evolution suggests that strong biases and constraints affect adaptive evolution, resulting in changes at a relatively small subset of available genes. Declines in the probability of gene reuse with increasing age suggest that these biases diverge with time.

## 1. Introduction

Parallel and convergent evolution of traits in independent populations inhabiting similar environments (‘repeated phenotypic evolution’) implicates natural selection [1–4]. Processes contributing to phenotypic evolution other than selection, such as mutation and drift, are unlikely to yield the same evolutionary shifts, again and again, in correlation with environment. Conversely, repeated use of the same underlying genes during parallel and convergent phenotypic evolution is thought to reflect biases and constraints on the supply and fixation of beneficial mutations. For example, some genes might contribute to adaptation more often than others because they have more standing genetic variation, higher mutation rates, larger effect sizes, more numerous beneficial mutations, fewer pleiotropic constraints, particular linkage relationships, or because they are involved in particular epistatic interactions with the genetic background [5–12]. Knowledge of these underlying effects and constraints might ultimately allow us to predict genetic evolution [5,8,9]. Instances of parallel and convergent phenotypic evolution provide an opportunity to measure the predictability of genetic changes underlying adaptation.

In some cases, high molecular specificity of a selective agent, such as a toxin in the diet that interferes with the function of particular proteins, drives repeated evolution in a small number of genes [7]. For example, resistance to tetrodotoxin in puffer fish and several snake species has repeatedly evolved by changes to a few amino acid residues in the outer pore of voltage-gated sodium channel proteins, where the neurotoxin binds to its target, causing paralysis [12,13]. This specificity explanation fails when many genes influence a trait, and changes to any one may produce similar alterations in phenotype. For example, all known cases of parallel reduction of complete armour plating in freshwater populations of threespine stickleback (*Gasterosteus aculeatus* species complex) involve the same major gene, *Eda* (*Ectodysplasin*) [14] (for other references see the electronic supplementary material, table S1), even though mutations in several genes of the *Eda*-signalling pathway in mammals are known to cause similar phenotypic changes in hair, teeth, sweat glands and dermal bones [15]. A ready supply of standing genetic variation in *Eda* in the ancestral population probably contributed to almost universal use of the same gene [14].

Despite a growing number of cases (reviewed in [7,8,10,16–19]), the probability of repeated use of the same genes in natural populations has not been estimated. The number of examples of repeated gene use in the published literature gives the impression that this probability might be high. Indeed, repeated use of the same genes is regarded as sufficiently common in evolution that the detection of equivalent genomic signatures of selection between independent natural populations that have adapted to similar environments is a valuable tool for discovering genes involved in adaptation [20–23]. Yet, the apparent frequency of reuse of genes might be distorted if biased methods are used to detect it or if less attention has been paid to cases in which different genes underlie repeated phenotypic evolution.

Here, we conducted an objective survey of the published literature to make a quantitative estimate of the probability of reuse of genes during repeated phenotypic evolution in independent lineages of natural populations. We clarify different approaches and biases that may affect estimation of this probability. We focus on repeated changes to the same gene, rather than on reuse of the same mutations, because the mutations are unknown in most cases. We include both protein-coding sequences and associated regulatory regions in our definition of a ‘gene’. We treat paralogous genes as different genes, which is a conservative decision because considering them to be the same gene increases the overall probability of gene reuse.

In addition, we test whether the probability of repeated use of the same genes declines as more distantly related taxa are compared. We would expect the probability to decline if phylogenetically distant taxa use different developmental pathways and networks more often than closely related species when they adapt to similar selection pressures [24]. Another reason to predict a decline is that pleiotropic constraints and the supply of beneficial mutations at a locus are likely to depend on its sequence and on its genetic background, both of which have had more time to diverge between taxa that are more distantly related.

Counterexamples are known in which repeated phenotypic evolution of closely related taxa used different genes, and in which distantly related taxa used the same genes [17]. Indeed, the frequency of such examples prompted Arendt & Reznick [17] to conclude that, from a genetic perspective, there is no clear distinction between ‘parallel evolution’ and ‘convergent evolution’. Yet, from a phylogenetic standpoint, it is useful to distinguish cases in which populations derived from the same or closely related ancestors evolved in the same direction (parallel evolution) from cases in which more distantly related, phenotypically differentiated populations evolved a similar trait (convergent evolution). This distinction is also reflected in the design of genetic studies reviewed here. Genetic studies of parallel phenotypic evolution compare multiple derived populations to the *same* ancestor (or to closely related populations representing their common ancestral state), whereas genetic studies of convergent evolution compare each of two or more distantly related, derived populations to *different* ancestral species, rather than to the common ancestor of all the taxa. Using this distinction, we ask whether the genetics of parallel and convergent evolution differ from one another in the probability of gene reuse.

## 2. On genomic approaches for detecting repeated genetic evolution

Genomic approaches hold great promise for detecting repeated use of the same underlying genes in phenotypic evolution. Counting the frequency of signatures of selection in the same genes between replicate natural populations adapting to similar environments, and exhibiting similar phenotypic changes, is straightforward in principle [20,22]. The approach has the advantage of broad coverage, and it allows the detection of mutations having relatively small effect sizes on fitness. For example, Jones *et al.* resequenced one individual from each of 10 marine (ancestral) and 10 phenotypically similar stream populations of threespine stickleback from around the northern hemisphere [22]. Stream and marine populations were consistently distinguished at about 200 loci, the outcome of repeated selection on standing genetic variation. Deeper sequencing of a single stream–marine pair found that 35 per cent of all genomic regions showing evidence of adaptive differentiation between the two populations also separated marine and stream populations globally. The probability of repeated use of the same genes was thus estimated as 0.35 in this study. Genome scans based on genetic markers rather than complete sequences also find evidence for parallel genetic evolution, but to different extents [25–30].

The main limitation of genomic studies is the lack of information on phenotypic traits affected by genes. Conceivably, separate mutations in the same genes might have divergent, rather than parallel effects on a phenotypic trait, or they might affect different traits. This problem can be partly overcome with functional experiments that determine if independent mutations in the same gene have the same phenotypic effects in different populations. It will be more difficult to identify those cases in which mutations in different genes lead to similar phenotypic effects. One might argue that fitness itself is the phenotypic trait addressed in genomic studies, since it evolves in parallel as replicate populations adapt to similar environments. On the other hand, fitness evolves in parallel even when phenotypes diverge, and hence estimates of the probability of gene reuse from genomic studies will not necessarily agree with estimates based on the identity of mapped genes underlying phenotypic traits evolving in parallel. For these reasons, it will eventually be interesting to compare results from the two approaches. Here, we chose to focus on genetic studies of repeated phenotypic evolution, which are presently more common than genome sequence comparisons of populations adapted to similar environments.

## 3. The genetics of repeated phenotypic evolution

We surveyed published genetic studies to estimate the probability of reuse of the same genes underlying repeated phenotypic evolution in natural populations. These studies used either of two main approaches to assess the genetic basis of phenotypic differences: genetic crosses and analysis of candidate genes.

Under a genetic cross approach, replicate populations that have independently evolved a particular phenotype are crossed to populations representing ancestral phenotypes. Quantitative trait locus (QTL) mapping methods are then used to test markers across the genome for an association with the phenotype of interest in hybrid offspring. Alternatively, mapping is carried out using admixed populations. Ideally, techniques such as fine mapping and functional assays are used subsequently to confirm gene identity (or at least to narrow the identified genomic region). The genetic cross approach has the advantage of genome-wide coverage, allowing multiple loci contributing to the derived phenotype to be discovered and the magnitude of their phenotypic effects to be estimated. For example, this approach was used to map repeated evolution of red wing patterning in *Heliconius erato* and *Heliconius melpomene* butterflies to the *optix* locus in both species [31–33].

More commonly, under this approach, researchers carried out genome-wide mapping in one pair of populations representing derived and ancestral phenotypes, and then other methods such as complementation crosses and localized (rather than genome-wide) mapping were used to determine whether the same gene or genomic regions were involved in other instances of repeated evolution. For instance, albinism was mapped to *Oca2* in two separate populations of cavefish, *Astyanax mexicanus*, whereas a complementation cross in a third population indicated that albinism arose through mutations in the same gene [34]. Or, genetic crosses were used to estimate the number of genes underlying a trait, followed by localized mapping of a candidate gene if Mendelian segregation was observed. For example, crosses between populations of the deer mouse, *Peromyscus maniculatus,* that have evolved on light and dark substrates revealed evidence for a single gene of major effect underlying a coat-colour phenotype [35]. Alleles of the candidate gene *Agouti* were found to segregate perfectly with the phenotype [18,35].

Under the alternative, candidate gene approach, one or a small number of designated genes is tested for association with phenotype. Ideally, the finding of such an association is accompanied by functional assays or other methods to confirm causality. This approach was used to demonstrate that electrical excitability of the myogenic electric organ, which has evolved independently in mormyroid and gymnotiform fishes, involved amino acid substitutions in the same functional regions of the sodium channel gene, *Scn4aa*, in both lineages [36,37]. The candidate gene approach determines whether ‘this same gene is involved’ when independent populations evolve a similar phenotype, but it does not provide estimates of the magnitude of the contributions of all genomic regions affecting a trait. The approach thus only provides a qualitative score of gene reuse. In turn, this may lead to higher estimates of the probability of parallel evolution compared with crossing and mapping studies. For this reason, we analyse data from the two approaches separately.

## 4. Literature search

We searched the literature for examples of repeated phenotypic evolution in natural populations that provided evidence on whether the same genes were used. To obtain a representative sample, we searched the online Thomson Reuters Web of Knowledge database for all studies in the subject area of evolutionary biology (as of 17 June 2012) that included the topic gene* and that contained either parallel* or converg* in the title (a ‘*’ at the end of a search term includes all words beginning with the preceding letters). We reasoned that these search terms would detect many studies that had tested the genetic basis of parallel or convergent phenotypic evolution regardless of outcome. In support, our search criteria detected multiple studies in which the genetic basis was found not to be the same between independent instances of repeated phenotypic evolution. In total, the search yielded 1612 publications, of which 25 met further criteria for inclusion in the study.

To be included, we required that a study addressed the genetic basis of a repeatedly evolved phenotype in natural populations rather than experimentally evolved or artificially selected populations. While the latter types of studies offer a wealth of information regarding parallel and convergent genetic evolution [38–40], our goal was to better understand repeated genetic evolution in wild populations, which span a greater range of ages and about which less is currently known. We included only studies with original data, rather than reviews. It was also necessary that the phenotypic trait in a study be an organismal-level trait rather than a molecular phenotype, since a protein sequence, expression pattern or function-based phenotype usually predetermines its underlying gene. We further required that repeated evolution in the phenotypic trait had been discovered prior to the discovery of its genetic basis. This was done to avoid an obvious bias accompanying a reverse discovery sequence in which the phenotypes were investigated only after repeated genetic changes had been found. Finally, we included only instances in which the direction of evolution was known or strongly suspected in independent populations, to exclude populations that might instead represent reversions to the ancestral state. This criterion meant that we could not include studies of the genetics of abdominal pigmentation in *Drosophila*, where the direction of evolution could not be verified [41], and the evolution of increased pigmentation in native American peoples, which involved evolution in the opposite direction compared with pigmentation changes in other human populations [42].

Data from the 25 papers meeting our criteria were arranged according to phylogeny of taxa and similarity of traits (see the electronic supplementary material, table S1). We then carried out an exhaustive search for all other publications on the same traits in the same species to ensure that we had the most up-to-date information on the genetic basis of the phenotypic traits in the examples originally identified by our objective search. We did not pursue citations found in papers included in our study that described other cases of parallel or convergent evolution not detected in our primary Web of Knowledge search. We felt that including them might produce a citation bias that would inflate the apparent probability of gene reuse. Adhering to this objective criterion forced us to leave out some well-known studies of the genetics of phenotypic evolution. For example, our primary search turned up three study systems in which repeated evolutionary loss of pigmentation involved the gene, *Mc1r*: beach mice [43], White Sands lizards [44] and Mexican cavefish [45]. However, the search did not turn up other known cases of pigmentation evolution involving *Mc1r* [46–51]. We stress that our aim was to estimate the probability of repeated genetic evolution, which demanded an impartial survey. We do not claim to have eliminated all sources of bias, especially publication bias and the difficulty of detecting and identifying genes of small effect.

With the help of TimeTree [52], we obtained phylogenies and node age estimates for all relevant taxa, including those from different study systems that had independently evolved a similar phenotype (see the electronic supplementary material, table S1). This allowed us to compare probability of gene reuse with estimated node age of common ancestors. In cases of parallel phenotypic evolution (i.e. independently derived forms are crossed, or compared, to the same recent ancestral form), node ages are also approximate times of onset of phenotypic divergence. In most cases of convergent evolution (i.e. different derived forms are compared with different ancestral forms), the onset of phenotypic divergence occurred within each lineage long after the divergence of the lineages themselves. Hence good estimates of the timing of phenotypic shifts [53] were often difficult to obtain, and we are unable to analyse the additional effects of trait origin times on the probability of gene reuse.

## 5. Calculating the probability of repeated gene use

**Figure 1.**
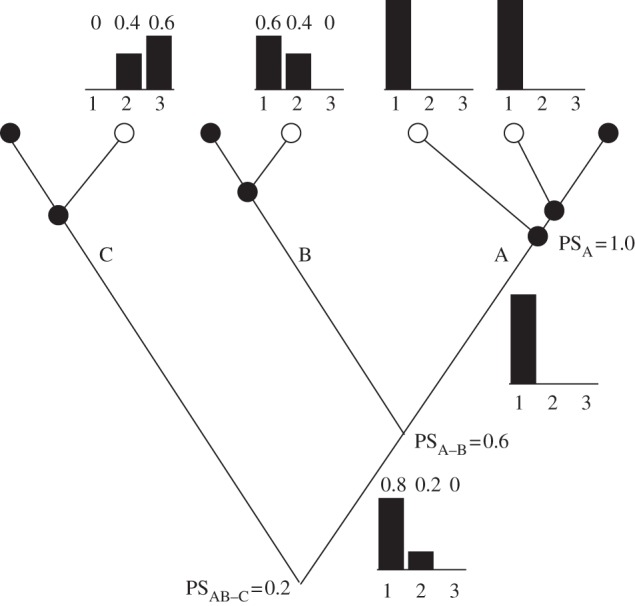
Hypothetical example to illustrate calculation of proportional similarity (PS) to measure probability of gene reuse between sister taxa. A, B, and C represent species having one or more populations that independently evolved a similar change in phenotype (open circles) compared with an ancestral phenotype (filled circles). Bar graph above each derived population indicates the relative contributions of each gene *i* to the phenotype (here, *i* is 1, 2 or 3). PS is calculated between a pair of taxa as PS = *Σ_i_* min(*p_i_*_1_, *p_i_*_2_), where *p_i_*_1_ and *p_i_*_2_ are the proportional contributions of gene *i* in the two taxa. Within a species, PS is measured between all pairs of derived populations and averaged. Relative contributions of genes are then averaged among populations (illustrated for species A by the bar graph immediately below node A). PS_A–B_ compares the relative contributions of the three genes in species B with the average for species A (PS = 0.6 + 0 + 0 = 0.6). PS_AB-C_ compares the relative contributions of the three genes in species C with the average of A and B, shown in the bar graph below the node connecting A and B (PS = 0 + 0.2 + 0 = 0.2).

We analysed data from genetic crosses and candidate gene studies separately. Studies that employed genetic cross methods provided effect sizes (per cent variance explained or magnitude of effect) that we used to compute the relative contribution of identified genes or QTL (if causal genes had not yet been identified) to the evolved phenotypic change in a particular cross. These effects were rescaled so that the contributions represented proportions and summed to 1.0 (see bar graphs at the tips of the hypothetical phylogenetic tree in figure 1). In a single case, effect sizes for two out of five mapped genes were not available, so the unexplained variance was split evenly between the two. Genes that were confirmed to have a major effect, either by complementation tests of shared use of a gene of major effect or localized mapping of a candidate gene in a cross in which the trait showed simple Mendelian segregation, were assigned an effect size of 1.

Probability of gene reuse between a pair of taxa was quantified using proportional similarity (PS) [54], calculated as PS = *Σ*_i_ min(*p_i_*_1_, *p_i_*_2_), where *p_i_*_1_ and *p_i_*_2_ are the proportional contributions of gene *i* in the two taxa (figure 1). This quantity treats the distribution of contributions by genes in each of the two taxa as a frequency distribution and measures their intersection. When causative genes within QTL were not known, co-localizing QTL were considered to represent repeated use of the same ‘gene’. It is possible that different genes within co-localizing QTL are responsible in different cases of repeated phenotypic evolution. However, at this point, QTL represent the best available information for many taxa. Future studies will be better able to estimate the prevalence of different but tightly linked genes underlying repeated phenotypic evolution.

When data were available on multiple derived populations for a given named species that independently evolved the same phenotype (e.g. two populations above the node for species A in figure 1), PS was calculated between all population pairs and then averaged, yielding a single PS estimate for the species. The species value for the relative contributions of different genes was then calculated by averaging the relative contributions of its multiple populations (see the bar graph below node A in figure 1). Our justification for using just one data point per species for a given trait was that frequency of gene use of derived populations within a species is expected to be greater than that between populations of different species, even after accounting for age differences. This is because populations within a species are typically crossed to the same ancestral form and so are not independent. They are also more likely to share standing genetic variation. This decision is conservative, because treating separate populations within species as independent replicates in the overall analysis raises the probability of repeated gene reuse. PS was then calculated separately between each sister pair in the phylogeny (e.g. PS is calculated between taxa A and B in figure 1). After calculating PS between two sister taxa at a given node, the relative contributions of genes in the two taxa were averaged (see the bar graph below the node connecting A and B in figure 1). This average was then used to calculate PS between sister taxa at the next node down the tree (e.g. between C and the node connecting taxa A and B in figure 1).

In our analysis of candidate gene studies, a given population or species received a score of 1 if use of the candidate gene was confirmed and a 0 if the assay used produced no evidence that the gene contributed to the trait. We recognize that assays were not always exhaustive and often could not completely rule out an effect of the gene on the trait. PS between two taxa was calculated as PS = min(*p*_1_, *p*_2_), where *p*_1_ and *p*_2_ are the proportional uses of the candidate gene in the two taxa. When data on multiple derived populations were available for a given named species, the species value for candidate gene reuse was calculated by averaging the values (0's and 1's) across the populations. PS was then calculated separately between each sister pair in the phylogeny. After calculating PS between two sister taxa at a given node, the proportional use values for candidate genes was averaged between the two taxa. This average value was then used to calculate PS between sister taxa at the next node down the tree. When more than one informative candidate gene was available at any given node, the above process was repeated for each gene and their PS values were averaged. Comparisons between two sister taxa, one of whose data were obtained using the candidate gene method, and the other of whose data came from genetic crosses, were included in the analysis of candidate genes. In such cases only, we treated the mapping data as though a candidate gene approach had been applied, assigning a score of 0 or 1 for candidate gene use. In some cases, the same node is used in both mapping and candidate gene analyses. However, the populations being compared in such cases are always mutually exclusive.

We repeated all analyses using a second, multiplicative measure of overlap of gene contributions between taxa, 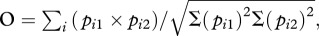 where *p_i_*_1_ and *p_i_*_2_ are the proportional contributions of gene *i* in the two taxa [55]. O represents the probability that a random draw from the proportional use distributions of each species results in the same gene, scaled so that the measurement is insensitive to the number of genes in the distribution. Applying this measure led to virtually identical results as PS, and so we present only PS. Throughout, standard errors and *p*-values should be regarded as heuristic because of the uncertainty about the degree of independence of observations in the meta-analysis.

## 6. Results

**Figure 2.**
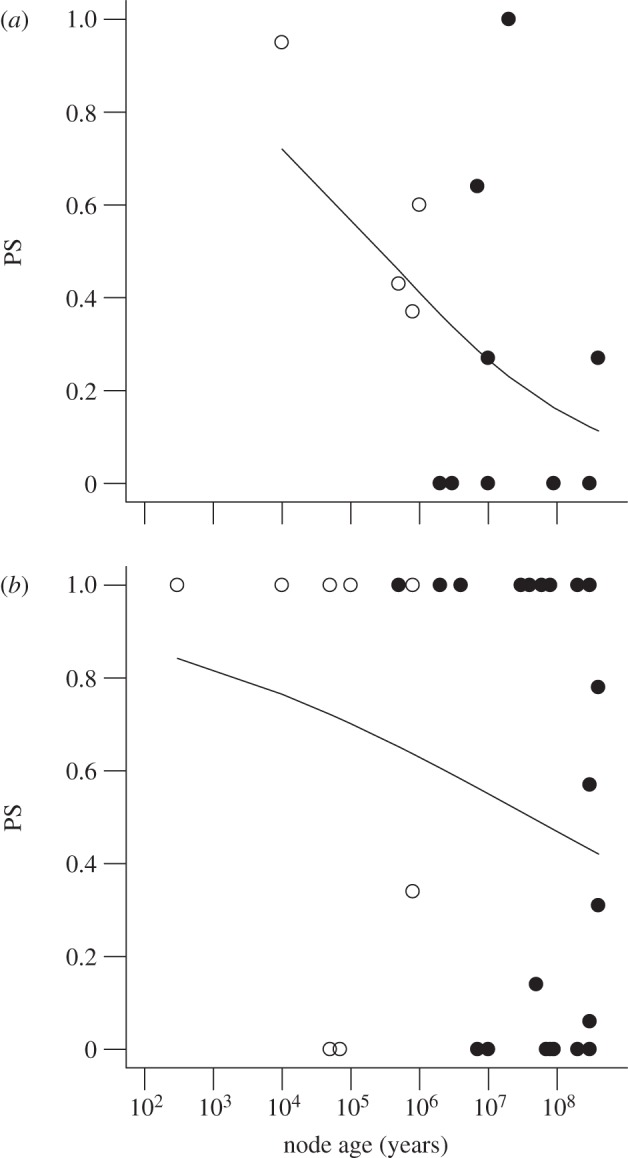
Measurements of the probability of gene reuse based on (*a*) data from genetic crosses and (*b*) candidate gene data. Open symbols represent average of PS between all pairs of derived populations within the same species (i.e. parallel evolution). Filled symbols represent similarity measurements between sister taxa at deeper nodes in the phylogenetic trees (i.e. convergent evolution). Curves are best-fit logistic regressions to the data.

Results are plotted separately for measurements based on genetic cross methods (figure 2*a*) and candidate gene methods (figure 2*b*). Each point represents the mean of PS between pairs of populations of a single species, if multiple populations were available (parallel evolution, open symbols), or between sister species or other sister taxa at deeper nodes of the phylogenetic trees (convergent evolution, filled symbols). Approximate ages of nodes are given along the horizontal axis. In the case of species values (open symbols), node age represents the approximate time at which repeated trait divergence began between ancestral and derived populations. Ages of sister species and more distantly related sister taxa (filled circles) only indicate the age of their common ancestor, since repeated phenotypic evolution typically occurred long afterwards (cf. figure 1).

On the basis of data from genetic crosses, estimated similarity of gene usage between taxa undergoing repeated phenotypic evolution in a trait is 0.32 ± 0.10 s.e. on average. The probability of gene reuse based on candidate gene data is 0.55 ± 0.08 s.e.

The results showed the predicted tendency for the probability of gene reuse between sister taxa to decline with the age of the node of their common ancestor (figure 2). Also, across the span of ages represented, estimated probability of gene reuse tended to be higher in candidate gene data than in data from genetic crosses. The predicted probability of gene reuse was high (around 0.8) in both datasets at the youngest nodes. This probability declined to about 0.10 by about 10^8^ years for mapping data, but remained higher (about 0.40) at the same node age for candidate gene data (figure 2).

To test these trends, we used logistic regression to model the relationship between PS, node age and genetic method (genetic cross versus candidate gene). Results indicate that the decline in the probability of gene reuse with node age is real (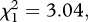 *p* = 0.04, one-tailed test). The effect of genetic method was not statistically significant in a two-tailed test (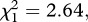 *p* = 0.10), and so these data do not fully resolve the difference between mapping and candidate gene data. These tests are conservative because many values of PS lie between 0 and 1 (figure 2), and so have lower residual variance than assumed by logistic regression. Reanalysis using quasi-binomial errors [56] slightly strengthen the above findings. Finally, a randomization test confirmed the overall correlation between PS and node age (*r* = −0.25, *p* = 0.04, one-tailed test).

Points obtained from comparing multiple populations within a species to the same ancestral form (open symbols in figure 2, representing parallel phenotypic evolution) are younger than points obtained by comparing species and higher taxa to different ancestors (filled symbols, representing cases of convergent phenotypic evolution). The decline in PS with older nodes thus implies that the probability of repeated use of the same underlying genes is indeed lower for convergent evolution than parallel evolution, as we defined these terms. Specifically, when estimated using data from genetic crosses, the probability of reuse of the same genes is 0.47 ± 0.15 s.e. on average in taxa undergoing parallel phenotypic evolution and 0.24 ± 0.12 s.e. on average in taxa undergoing convergent phenotypic evolution. Likewise, using candidate gene data, the probabilities of reuse of the same genes are 0.67 ± 0.17 s.e. and 0.51 ± 0.09 s.e. on average for parallel and convergent phenotypic evolution, respectively.

## 7. Discussion

Our results based on data from genetic crosses indicate that when similar traits evolve independently in different lineages, the probability that the same genes are used is estimated to be 0.32, on average. The probability estimated from candidate gene studies is 0.55, on average. One explanation for such a high probability of repeated use of the same genes is that at the time of adaptation, effect sizes and availability of beneficial mutations were strongly biased towards a small number of genes. This result can be summarized by the ‘effective’ number of genes, equivalent to the number of loci available if all have effects of equal magnitude and the same probability of fixation. For example, imagine that in two ancestral populations there are *n* equivalent genes underlying a trait under selection in which new advantageous mutations might occur and fix. If a major effect mutation occurs and fixes in one gene in one of the populations, the probability is 1/*n* that a second population experiencing similar selection fixes a mutation in the same gene. In this case, a probability of gene reuse of 0.32 corresponds to an *n* of 1/0.32 = 3.1 effective genes. A probability of gene reuse of 0.55 corresponds to an *n* of 1/0.55 = 1.8 effective genes. This rough calculation is simplistic, because real genes do not have equivalent effects. In addition, it does not indicate the cumulative number of genes that might contribute if parallel or convergent evolution is repeated many times. Nevertheless, the high probabilities of gene reuse estimated from published data indicate that the effective number of genes used in parallel and convergent phenotypic adaptation is typically small. If the causes of this low number can be elucidated, then genetic evolution may indeed be somewhat predictable [7,8,11].

It is difficult to judge how surprising these estimates of effective number of genes are without knowing the total number of genes available in which mutations would cause similar phenotypic changes. Some data are available to assess this. Of the six genes of the *Eda-*signalling pathway, mutations in most of which produce a similar phenotype in mammals, only two have been found to be associated with lateral plate variation in threespine stickleback: *Eda* and the receptor *Edar* [15]. Similarly, Streisfeld & Rausher [11] noted that changes to any of the nine enzymes of the anthocyanin biosynthetic pathway would alter pathway flux and produce a change in intensity of flower pigmentation. In accord, 37 spontaneous mutations affecting floral pigment intensity have been detected in five of these nine genes, predominantly in coding regions (another 32 mutations affecting floral pigment intensity occurred in transcription factors). However, in all seven cases in which evolved differences in pigment intensity were mapped, the fixed changes mapped to transcription factors that regulate the pathway genes rather than to the genes themselves, indicating a strong fixation bias away from coding mutations in pathway proteins [11]. In five of these seven cases, the changes occurred in a gene encoding an *R2R3 Myb* transcription factor. Such findings suggest that the number of genes used and reused in adaptive evolution is a small subset of available genes. A host of factors may lead to much higher probabilities of certain genes being involved in phenotypic adaptation than others, including amounts of standing genetic variation, differences in mutation rates or mutation effect sizes, pleiotropic constraints, linkage relationships and epistatic interactions with the genetic background [5–12].

Any explanations for such a high probability of repeated use of the same genes must also explain why this probability declines as more distantly related taxa are compared. First, the high probability of repeated use of the same genes by young, closely related populations might result in part because they have access to the same pool of standing genetic variation [14,57], an option not available to more distantly related taxa. Second, as lineages diverge, not only do the specific genes that affect the phenotypic trait diverge in sequence, but the genetic backgrounds with which they interact diverge as well. Hence, the biases that favour use of some genes over others during repeated phenotypic evolution themselves should evolve, in which case we would expect the probability of repeated use of the same genes to decline with time and genetic divergence. The probability that changes to the same genes produce similar phenotypic changes is also likely to be reduced the more widely divergent the lineages [58], unless gene functions are highly conserved.

Repeated evolution can be divided into two types: parallel evolution, whereby evolution begins from the same starting point, and convergent evolution, whereby evolution begins at different starting points. Arendt & Reznick [17] argued that from a genetic perspective there is no clear distinction between parallel and convergent evolution. We found that average PS of genes underlying parallel phenotypic evolution was greater than that underlying convergent evolution (figure 2). The reasons are probably similar to those described for the effect of node age, since points representing parallel evolution have younger node ages than points representing convergent evolution. If evolution is biased towards some genes over others, populations beginning from the same ancestral genome will more likely share these biases than populations beginning from divergent genomes. However, there is no sudden break in the probability of gene reuse between parallel and convergent evolution (figure 2). The distinction is one of degree rather than of kind.

Our estimates based on candidate genes are higher than those based on genetic crosses. Although not statistically significant in our analysis, the difference suggests that the calculated probability of repeated use of the same gene depends on the methods used to detect it. If the difference is real, what are the possible reasons? Whereas genetic cross methods allow us to estimate the contributions of all genes (or at least all genes of moderate to major effect) to repeated phenotypic evolution, the candidate gene approach allows us to determine only whether a specific gene of interest makes a contribution in each case. This essentially lowers the bar for a positive outcome in the case of candidate genes, because the probability of reuse of a gene of interest between two taxa is likely to be higher than the proportional shared use of genes when all mapped genes are considered. Another reason is that the candidate gene method might be more strongly affected by publication bias than estimates based on genetic crosses. We suspect that studies which fail to confirm a role for a candidate gene are more likely to go unreported than results from mapping studies, which produce noteworthy findings if evidence for genes is found anywhere in the genome. On the other hand, estimates of PS that take magnitude of effect into account (i.e. those based on genetic crosses) are prone to higher sampling error, which will tend to cause a downward bias in estimates for the probability of gene reuse.

Caution is warranted when interpreting our results because of numerous judgements and uncertainties inherent to a meta-analysis involving heterogeneous data collected from various organisms, traits and genes. In some cases, we considered overlapping QTL to be reuse of the same ‘gene’, although we may eventually learn that different genes within the QTL underlie repeated phenotypic evolution. While this and other factors already described, such as publication bias, may cause us to overestimate the probability of gene reuse, still other factors may cause an underestimation. For example, our definition of repeated genetic evolution treats paralogous genes as different (several examples were present in our dataset, including the paralogous genetic basis of convergent evolution of caerulein skin toxin in frogs [59], of digestion of foregut-fermenting bacteria in leaf-eating colobine monkeys and ruminant artiodactyls [58,60], and of red flower colour in *Mimulus* spp. [61,62]). In addition, multiple populations within a single named species were represented by only a single data point in our analysis (the average) to prevent rampant parallel genetic evolution within any one species from unduly affecting the results. Finally, the number of studies on which we have based our analyses is not large, which also adds uncertainty to these results. Despite these uncertainties, our aim here has been to stimulate thinking about these issues and to move towards a quantitative understanding of repeated genetic evolution, which we have attempted with the best available information.

As we accumulate more studies of the genetics underlying repeated phenotypic evolution in natural populations, we will be better able to estimate the probability of the same genes being used. In turn, this will enhance our ability to ask what factors explain variability in genetic parallelism and convergence. For example, broader sampling may allow us to ask whether there is a difference in probability of gene reuse between loss-of-function and gain-of-function traits, or between genes of major and minor effect. Improved knowledge of the biochemical functions and pathway positions of genes will allow us to address whether genes that influence a greater number of other genes in developmental pathways are more or less likely to underlie repeated phenotypic evolution than genes acting at terminal points in the pathways [63]. Knowledge of mutations will allow us to address how properties such as dominance contribute to the probability they will repeatedly underlie evolution of a phenotype [44]. Further tests are required of the mechanisms proposed to underlie the high rate of reuse of the same genes, such as pleiotropy and mutation bias [11]. In the future, it will be interesting to compare our estimates with probabilities of gene reuse from whole-genome sequences of populations adapting to similar environments. We feel that studies starting from purely genetic and genomic approaches must incorporate steps to understand the phenotypic effects of the genetic changes detected. This will be important to determine whether parallel genomic signatures resulted from selection on the same phenotypic traits in different populations, and to determine the mechanisms of selection. Likewise, studies of phenotypic evolution should be followed through to its genetic basis to gain a better understanding of the consequences of repeated phenotypic evolution at the level of genes and mutations. With solid connections between phenotypes and genotypes, repeated phenotypic evolution provides a powerful way to study the predictability of genetic changes underlying adaptive evolution.
